# Ascorbic acid synthesis and transportation capacity in old laying hens and the effects of dietary supplementation with ascorbic acid

**DOI:** 10.1186/s40104-018-0284-7

**Published:** 2018-10-01

**Authors:** Liping Gan, Hao Fan, Wei Nie, Yuming Guo

**Affiliations:** The State Key Laboratory of Animal Nutrition, College of Animal Science and Technology, China Agricultural University, Beijing, 100193 People’s Republic of China

**Keywords:** Ascorbic acid, Antioxidant capacity, Immunity, L-gulonolactone oxidase, Old laying hens

## Abstract

**Background:**

Laying hens over 75 weeks of age commonly show great declines in immunity and production performance. It is unclear whether these declines can be relieved by supplementing with ascorbic acid (AA) in feed. Two trials were conducted to investigate the synthesis and metabolism of AA in layers of different ages and the effects of dietary supplemental AA on the performance and the immune and antioxidant statuses of 78 weeks old hens.

**Methods:**

In Exp. 1, equal numbers (24 hens) of 35 weeks old (Young) and 75 weeks old (Old) layers were fed the same diet without AA supplementation for 4 weeks. In Exp. 2, 360 healthy 78 weeks old laying hens were randomly assigned to 4 treatments (basal diet supplemented with 0, 0.25, 0.5, or 1 g AA/kg diet) in an 8-week feeding trial.

**Results:**

The old hens tended to have decreased L-gulonolactone oxidase (GLO) synthase activity in the kidney and liver than that of the young hens (*P* = 0.07 and *P* = 0.05, respectively). Compared with the young hens, the old hens had lower hepatic antioxidant capacity allowing for the lower thioredoxin (*TXN*), thioredoxin reductase (*TXNR*) and cytochrome b5 reductase (*CYB5R*) gene expression (*P* < 0.05), whereas increased sodium-dependent vitamin C transporter (*SVCT*) 1 expression levels in the ileum and kidney and enhanced splenic and hepatic AA concentrations (*P* < 0.05). Dietary supplementation with AA significantly decreased GLO enzyme activity but increased splenic AA concentration and anti-bovine serum albumin IgG levels (*P* < 0.05) and tended to increase CD4^+^ T lymphocyte numbers (*P* = 0.06) in serum. Supplementation of 0.25 g AA/kg diet significantly increased hepatic total antioxidant capacity (T-AOC, *P* < 0.05) relative to the control group.

**Conclusions:**

Laying hens could synthesize AA in both the kidney and the liver, though the GLO enzyme activities were 100 times greater in kidneys than in livers. The old laying hens had greater absorption and reabsorption capacity and higher AA retention in some tissues that did the young hens. Dietary supplementation of AA can improve the health of old layers by enhancing immunity and antioxidant capacity.

## Background

Maintaining excellent immune status, production performance, and egg quality in old laying hens (i.e. until 80 to 100 weeks of age) is an ongoing interest in poultry production [1]. In general, laying rate and egg quality begin to decline in layers after 60 weeks of age, with larger numbers of abnormal eggs produced and increased breakage rates. These declines cause substantial economic losses [2]. Thus, it is important to develop nutritional solutions to mitigate the decrease in laying rate in old layers.

Some previous studies have suggested that the decline in laying rate in old layers is related to oxidant stress and decreased immunity [2–4]. Ascorbic acid (AA) is a potent antioxidant that is usually supplemented in feed to alleviate oxidative stress and improve immunity [5, 6]. After a cascade of two-round oxidation and the loss of two electrons, the oxidized form of AA, termed dehydroascorbic acid (DHA), is formed. Poultry have the capacity to synthesize AA in the kidney through the glucuronatexylulose-xylulose cycle in the presence of L-gulonolactone oxidase ([EC 1.1.3.8], GLO). All AAs are transported to cells by sodium-dependent vitamin C transporter 1 (SVCT1) and sodium-dependent vitamin C transporter 2 (SVCT2), while DHA enters and leaves cells via facilitated glucose transporters [7–9]. The AA requirements of laying hens are met in two ways: synthesis in vivo (endogenous) and absorption from feed (exogenous). Dietary supplementation with AA improved the production performance and immunity status of old laying hens (72 weeks old) subjected to severe heat stress conditions [10]. However, some results have shown that the supplementation of AA was found to have no effect on the production performance of laying hens (31 weeks old) under oxidant stress [11] and broiler breeder chickens [12]. Such inconsistent results regarding AA supplementation prompted an examination of the changes in distribution and transportation of AA in different tissues and GLO enzyme activity in poultry of different ages. In poultry production, old layers (over 75 weeks of age) that are subjected to a variety of stresses generally present decreases in immunity and laying rate. Few studies have focused on the effects of AA supplementation on immune function, antioxidant status, and production performance in old layers. This information is essential for understanding AA metabolism and developing strategies for sustaining optimum performance in old laying hens. We hypothesized that old laying hens have lower abilities than do young hens to synthesize and absorb AA and that dietary supplementation with AA can improve AA content in tissues, antioxidant state, and production performance. Two consecutive trials were carried out to ascertain 1) the ability of old laying hens to synthesize, absorb, transport, and utilize AA and 2) the effects of dietary supplementation with AA on the production performance, egg quality, antioxidant status, and immunity of old laying hens.

## Methods

### Animals, experimental design, and diets

#### Exp. 1

A total of 48 healthy commercial Hy-line Brown laying hens (35 and 75 weeks of age, 24 of each group) of similar weight were used in a controlled trial (young hens vs. old hens). All of the birds were raised in cages, with two birds per cage, and were fed the same standard commercial layer diet without AA supplementation for 4 weeks before sample collection. Feed and fresh water were available ad libitum. Twelve hens were randomly selected from each group and sacrificed using pentobarbital anaesthesia. The same position from the left side of the kidney, along with the liver, spleen, brain, shell gland, and ovary, were sampled, wrapped in aluminium foil, immediately frozen in liquid N_2_, and then maintained at − 80 °C for further analysis. Then, the mucosae from the duodenal, jejuna, and ileal segments were collected and rapidly frozen in liquid N_2_ and preserved at − 80 °C until analysis.

#### Exp. 2

A total of 360 healthy, 78 weeks old laying hens with similar performance were randomly distributed among 120 cages, with three hens per cage. All hens were randomly assigned to four dietary treatments, with 6 replicates of 15 hens each (90 hens per treatment). Five cages of 15 layers were considered to compose one replicate unit. One group was fed the basal diet only (control). The remaining three groups were fed one of the AA-supplemented diets (supplemented AA at a dose of 0.25, 0.5, or 1 g AA/kg diet) over the 8-week feeding trial.

Batches of the experimental diets were produced every 4 weeks to limit the loss of AA activity during feed storage. The composition and nutrient level of the basal diet are shown in Table 1. All of the hens had free access to water and feed during the 8-week trial. The hens were housed in an environmentally controlled room maintained at 24 °C and had a daily lighting schedule of 16 h light and 8 h dark. At the end of the experiment, six layers were randomly selected from each treatment before laying eggs in the morning and sacrificed with pentobarbital anaesthesia. The kidney, spleen, and liver of the laying hens were sampled, wrapped in aluminium foil, frozen in liquid N_2_, and maintained at − 80 °C until analysis.

**Table 1:** Composition and nutrient level of the basal diet

Items	Content, g/kg	Nutrient level	
Corn	596	AME, MJ/kg	10.9
Soybean meal	236	Crude protein, g/kg	155
Wheat	41.3	Lysine, g/kg	7.4
Soybean oil	10.0	Methionine, g/kg	3.3
Calcium carbonate	95.0	Calcium, g/kg	40
Calcium phosphate	14.0	Available phosphorus, g/kg	3.4
*DL*-methionine	0.8	Total phosphorus, g/kg	5.8
Vitamin premix^a^	0.2		
Mineral premix^b^	2.0		
Sodium chloride	3.0		
Choline chloride	1.5		
Antioxidants	0.2		

^a^Provided per kilogram of diet: vitamin A, 10,000 IU; vitamin D_3_, 2,400 IU; vitamin E, 40 IU; vitamin K_3_, 2 mg; vitamin B_1_, 2 mg; vitamin B_2_, 6.4 mg; vitamin B_6_, 3 mg; vitamin B_12_, 0.02 mg; folic acid, 1 mg; niacin, 30 mg; Ca-pantothenate acid, 10 mg

^b^Provided per kilogram of diet: Cu, 8 mg; Zn, 75 mg; Fe, 80 mg; Mn, 100 mg; Se, 0.15 mg; I, 0.35 mg

### Production performance and egg quality (Exp. 2)

The production performance of the laying hens was measured from 78 to 85 weeks of age. Daily egg production and egg weight were determined per replicate unit. Abnormal eggs, including soft-shelled, cracked, and broken samples, were also recorded daily. Feed intake was measured on a weekly basis. The feed conversion ratio (FCR, feed intake/egg weight) was calculated from egg production and feed intake. At 4-week intervals and at the end of the experiment, eggs from each treatment were selected for quality analysis. Eggshell strength, egg weight, egg yolk colour, Haugh unit, and albumen height were tested using a digital egg tester (NABEL, DET-6000). Eggshell thickness was measured at the large end, equatorial region, and small end using an Eggshell Thickness Gauge.

### Antibody response of immunoglobulin G against bovine serum albumin (Exp. 2)

One bird (82 weeks of age) from each replicate was immunized with 1 mL 1% bovine serum albumin (BSA). Blood samples (1.5 mL) were collected from the wing veins of the immunized birds on the 3^rd^, 6^th^, 10^th^, and 14^th^ day after immunization. The detection of specific systemic antibody response of immunoglobulin G (IgG) against BSA in serum was performed by indirect enzyme-linked immunosorbent assay as described by Alizadeh et al. [13].

### Flow cytometric analysis of the classification of T lymphocytes (Exp. 2)

Peripheral blood mononuclear cells (PBMCs) were isolated using a Ficoll density centrifugation [14]. Briefly, heparinized blood was diluted with Hank’s balanced salt solution at a ratio of 1:1 (no calcium and no magnesium, Life Technologies, Burlington, Vermont, USA) and was carefully layered on top of Histopaque 1077 (Sigma-Aldrich Corporation, Burlington, Vermont, USA) in a 10-mL centrifuge tube at a 2:1 ratio. After centrifugation for 30 min at 3,000 r/min at 20 °C, the PBMCs at the plasma–Ficoll interface were collected and then washed three times with cold RPMI-1640 medium. Lymphocytes were stained with anti-CD3-SPRD, anti-CD4-FITC and anti-CD8-PE for 30 min on ice. All staining was performed using 0.1 μg of each antibody for 100,000 cells. After that, cells were washed and resuspended in PBS. Propidium iodide was added to cells, and portion of CD4^+^ and CD8^+^ T lymphocytes were analysed by flow cytometry (Navios, Beckman-CouIter, USA).

### Measurement of ascorbic acid and dehydroascorbic acid (Exp. 1 and 2)

Ascorbic acid was measured using a high-performance liquid chromatography (HPLC)-ultraviolet (UV) light detection method as described by Robitaille et al. [15] and Amano et al. [16], with some modifications. The spleen, liver, kidney, brain, shell gland, and ovary tissue samples in Exp. 1 and the spleen tissues in Exp. 2 (approximately 100–120 mg) were homogenized in 1.6 mL of 5.4% metaphosphoric acid and centrifuged at 16,000 × *g* for 15 min at 4 °C. The centrifugal supernatants were divided into two aliquots of 600 μL in 1.5 mL plastic Eppendorf tubes. For the measurement of total AA (AA plus DHA), an equal volume of 5 mmol/L Tris (2-carboxy ethyl) phosphine hydrochloride (TCEP) in water (pH 2) was added. Then, the samples were allowed to react for 2 h at 4 °C in the dark. To measure AA, an equal volume of water was added instead of TCEP, and the samples were maintained on ice in the dark. Both samples were then centrifuged at 12,000 × *g* for 10 min at 4 °C. Next, the resultant supernatants were transferred to auto-injection vials and injected immediately onto the HPLC column (10 μL injection volume) for further analysis.

The analysis was carried out using a Waters 2695 Separations Module equipped with a Waters 717 plus Autosampler and a Waters 2487 dual wavelength UV detector set to 246 nm. The column was a reverse-phase Dikma Diamonsil C18 (4.6 mm × 150 mm, 5 μm) fitted with a Dikma Diamonsil C18 guard column. The column temperature was maintained at 25 °C. The mobile phase consisted of 50 mmol/L KH_2_PO_4_ (pH 2.8), 1 mmol/L EDTA, and 5% methanol at a flow rate of 0.8 mL/min. The retention time was 3.89 min. A 7-min delay was provided between injections to produce a smooth baseline. For both methods, a standard curve was developed by using a peak area linear regression equation from six AA standards made in metaphosphoric acid and EDTA ranging in concentration from 2 to 50 mg/L.

### Measurement of L-gulonolactone oxidase activity (Exp. 1 and 2)

L-gulonolactone oxidase enzyme activity was measured by determining the rate of total AA synthesized in liver or kidney tissues by adding the substrate (L-gulonolactone). Maximum GLO synthesis in chick kidney was attained after supplementation of 5 mmol/L of L-gulonolactone [17]. The procedures of Hooper et al. [18] and Ching et al. [19] were followed, with some modifications. Approximately 100 mg of liver or kidney sample was homogenized in 1.6 mL 50 mmol/L sodium phosphate buffer (PB) at pH 7.4 containing 0.2% sodium deoxycholate (TCI, Shanghai, China) and 1 mmol/L EDTA and centrifuged at 20,000 × *g* for 30 min at 4 °C. The supernatants were divided into 2 aliquots of 400 μL in 5-mL plastic tubes. To detect GLO activity, 200 μL 50 mmol/L L-gulonolactone and 1,400 μL PB were added to one of the aliquots, mixed, and incubated in water for 30 min at 37 °C in the dark. A blank was run with each sample to correct for endogenous AA. Phosphate buffer (1,600 μL) was added to the supernatants to a total volume of 2 mL. The resulting solution was incubated in water for 30 min at 37 °C in the dark. The reaction was stopped with 2 mL 5% trichloroacetic acid (TCI, Shanghai, China). Then, the mixture was incubated in the dark for 20 min at room temperature and centrifuged at 4 °C, 10,000 × *g* for 5 min. The supernatants were used to detect GLO-synthesized AA. The procedure for measuring AA was as described above.

### Quantitative real-time polymerase chain reaction (qRT-PCR) analysis (Exp. 1)

Total RNA was isolated from the liver, spleen, kidney, and intestinal samples using TRIzol reagent (TAKARA, Dalian, Laoning, China) according to the manufacturer’s protocol. The cDNA synthesis was performed using a PrimeScript RT reagent kit with gDNA eraser (TaKaRa, Dalian, Liaoning, China) according to the manufacturer’s instructions. The primer sequences for the target and reference genes are shown in Table 2. All of the measurements were performed in triplicate, and the average values were calculated. Relative mRNA expression levels of *GLO*, *SVCT1*, *SVCT2*, thioredoxin (*TXN*), thioredoxin reductase (*TXNR*), and cytochrome b5 reductase (*CYB5R*) genes were normalized to the expression of the housekeeping gene *GAPDH* using the 2^–ΔΔCt^ method [20, 21]. The products were separated on 1.2% agarose gel and stained with Gelred.

**Table 2:** Primer sequences of housekeeping and target genes

Genes		Prime sequence^a^	NCBI number	Product size, bp
*GAPDH*	F^b^	GACCCCAGCAACATCAAATG	NM_204305.1	110
	R	TTAGCACCACCCTTCAGATG		
*GLO*	F	TCTCCTCTGGATCAGCACCT	XM_015285218.1	131
	R	AGCGGCACTCGTAGTTGAAG		
*SVCT1*	F	GGGATACCCACGGTGACCTC	XM_004944768.2	100
	R	GCCGTGCACAGGAGTAGTAA		
*SVCT2*	F	TGTCTTGTGCTCCTCCTCCT	NM_001145227.1	101
	R	TCCATTCCCTGTCCCAAATA		
*GLUT1*	F	TAGTACTGGAGCAGGTGGCAGA	NM_205209.1	124
	R	CGGCACAAGAATGGATGAAA		
*GLUT3*	F	TGCTGATAATTGGGCGCTTC	NM_205511.1	150
	R	CCACCAGGATGCCTACAACT		
*TXN*	F	GATTTCTCTGCCACATGGTGT	NM_205453.1	117
	R	ATCTTGGGCATCATCCACAT		
*TXNR*	F	GCTTCCTATGTTGCCTTGGA	NM_001030762.2	112
	R	TGTTTGCCATATCCTGGTCA		
*CYB5R*	F	GTGGATCACGTTCTGGGTCT	NM_001291805.1	119
	R	ACGAAGCCCTTGTCATCATC		

^a^ Primer sequences are displayed in the 5′ → 3′ direction

^b^ *F* forward primer, *R* reverse primer

### Antioxidant activity assay (Exp. 1 and 2)

In Exp. 1, the gene expression levels of *TXN*, *TXNR*, and *CYB5R* in the liver were tested using qPCR as described above. The GSH and GSSG contents in the liver (both experiments) were detected using GSH/GSSG reagent kits (Beyotime, S0053, Shanghai, China) according to the manufacturer’s guidelines. In Exp. 2, total antioxidant capacity (T-AOC) and malondialdehyde content in the liver were detected by a biochemical method following the instructions provided with the reagent kits (T-AOC, A015; malondialdehyde, A003) purchased from Nanjing Jiancheng Bioengineering Institute of China.

### Statistical analysis

In Exp. 1, the significance of differences between experimental groups was assessed by independent sample *t*-tests performed with SPSS statistical software (SPSS for Windows, version 22; IBM). All of the data in Exp. 2 were analysed using One-way ANOVA (SPSS for Windows, version 22; IBM). Linear and quadratic polynomial contrasts were used to evaluate the effects of the different dietary levels of AA. Statistical differences were considered significant at *P* < 0.05, and 0.05 < *P* < 0.10 was viewed as a trend towards significance.

## Results

### L-gulonolactone oxidase gene expression and enzyme activity in liver and kidney tissues

**Fig. 1:**
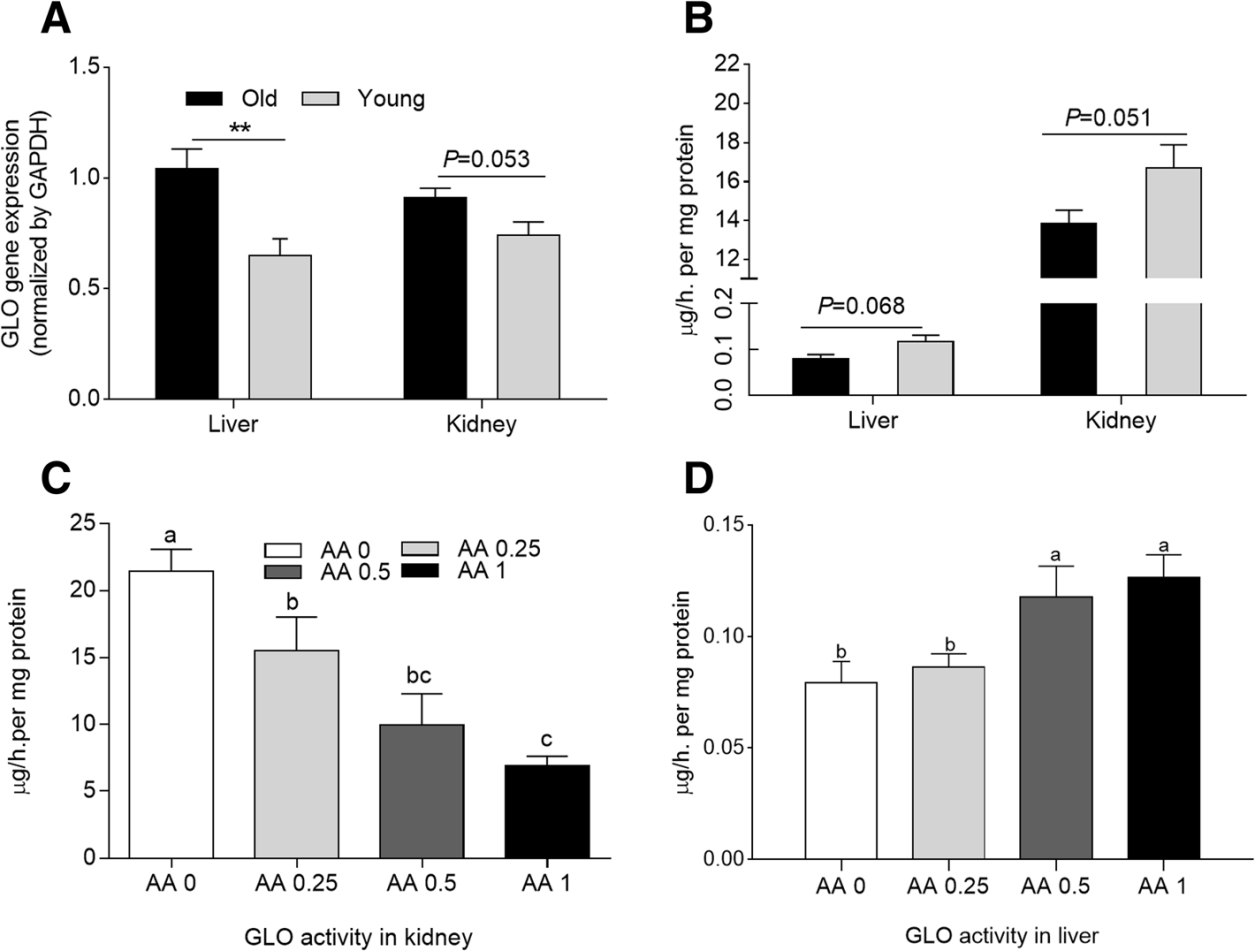
*GLO* gene expression and activities in kidney and liver. **a** *GLO* gene expression and **b** GLO enzyme activity in the kidney and liver of 35-week-old laying hens (Young) and 75-week-old laying hens (Old) in Exp. 1. *means *P* < 0.05. **c-d** Effects of dietary supplementation of AA on GLO activity in the kidney and liver of the old laying hens in Exp. 2. Within each panel, means without a common letter differ at *P* < 0.05

In Exp. 1 as shown in Fig. 1a and b, *GLO* gene expression in the liver and kidney was higher in the old hens than in the young layers (*P* < 0.05 and *P* = 0.05, respectively). However, GLO activity in the livers and kidneys of the old layers showed a decreased tendency compared with the corresponding activity in young birds (*P* = 0.07 and 0.05, respectively). In Exp. 2, regardless of concentration, dietary supplementation with AA significantly reduced GLO activity in the kidneys of old laying hens (*P* < 0.05). Moreover, GLO activity in the kidney decreased linearly with increasing AA supplementation (Fig. 1c). A small amount of AA was synthesized in the liver of old laying hens in Exp. 2 (0~ 0.12 μg/h per mg protein). The addition of 0.5 and 1 g AA/kg diet significantly enhanced GLO enzyme activity in the liver of old hens (Fig. 1d).

### Ascorbic acid and dehydroascorbic acid contents in different tissues

**Fig. 2:**
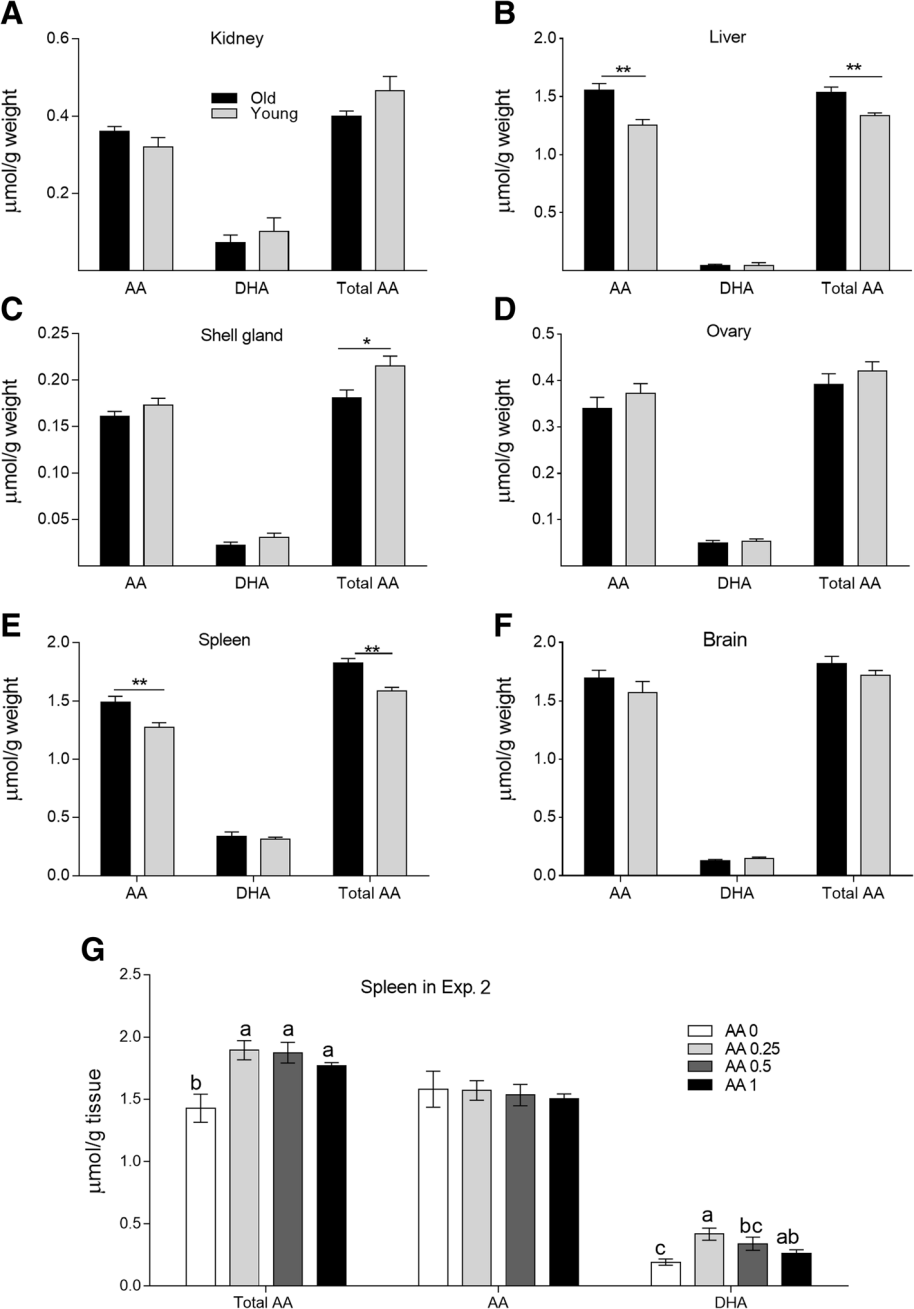
AA contents in different tissues. **a–f** Total AA, AA, and DHA contents in the kidney, liver, shell gland, ovary, spleen and brain of the old and young layers in Exp. 1. *means *P* < 0.05; **means *P* < 0.01. **g** Effects of dietary supplementation of AA on splenic total AA, AA, and DHA concentrations of old laying hens in Exp. 2. Within each panel, means without a common letter differ at *P* < 0.05

The concentrations of AA, DHA, and total AA (both AA and DHA) in different tissues are presented in Fig. 2. Old hens had higher concentrations of AA and total AA in the liver and spleen than did young hens (*P* < 0.05). In contrast, the young layers had higher AA retention in the shell gland than did old layers (*P* < 0.05). However, the levels of AA in the brain and kidney were similar (*P* > 0.05) between the young and old layers. In Exp. 2, irrespective of the amount of AA supplemented in the diet, AA supplementation significantly enhanced (*P* < 0.05) total AA concentration in the spleen relative to that in the control treatment. However, AA content did not differ among the treatments. Dehydroascorbic acid contents tended to decrease as the amount of AA supplementation increased. Supplementation with 0.25 and 0.5 g AA/kg diet significantly increased DHA content in the spleen relative to that of the control treatment (*P* < 0.05, Fig. 2g).

### Relative mRNA expression of ascorbic acid and dehydroascorbic acid transporters in different tissues

**Fig. 3:**
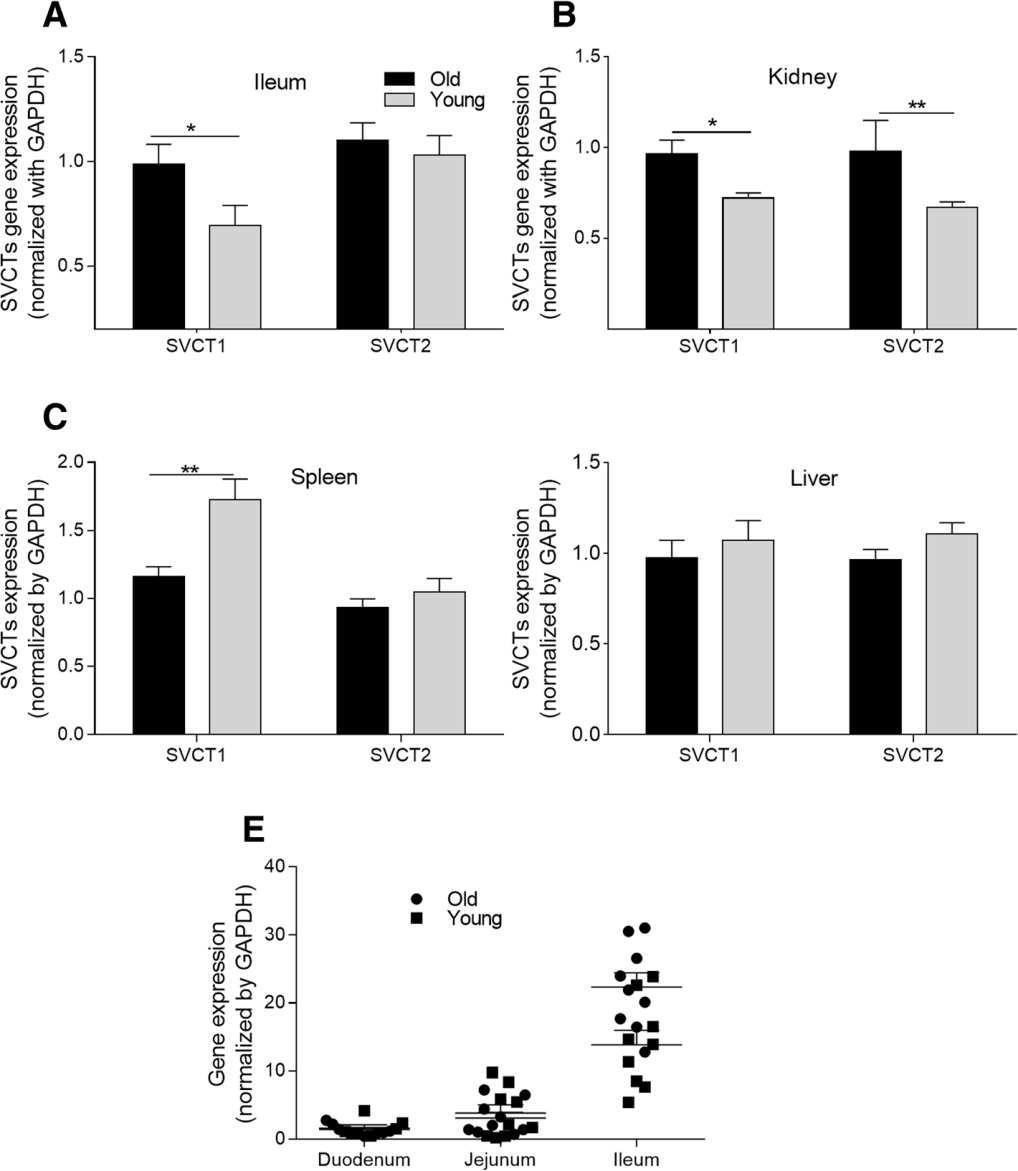
Transporter gene expression levels of the old and young hens in Exp. 1. **a-d** Sodium dependent vitamin C transporter (*SVCT*) 1 and *SVCT2* gene expression levels in the ileum, kidney, spleen and liver of old hens (Old) and young hens (Young). **e** The *SVCT1* gene expression levels in the duodenum, jejunum, and ileum. *means *P* < 0.05; **means *P* < 0.01

The expression levels of AA transporter genes of *SVCT1* and *SVCT2* in the liver, kidney, spleen, and intestinal segments are shown in Fig. 3. The *SVCT1* expression in the ileum was markedly higher in the old hens than in the young hens (*P* < 0.05, Fig. 3a). In the small intestine, *SVCT1* had the highest expression level in the ileum. There were no differences in *SVCT*s gene expression levels in the duodenum and jejunum (data not shown). In addition, old hens had significantly higher gene expression of *SVCT1* and *SVCT2* in the kidney than did young hens (*P* < 0.05). The *SVCT*s gene expression levels in the spleen and liver did not significantly differ between the old and young hens. The young hens had elevated *SVCT1* expression (*P* < 0.05) in their spleens. No significant difference in *SVCT2* expression was observed among the old hens. In addition, no differences between the young and old hens were observed in hepatic *SVCT1* and *SVCT2* gene expression (data not shown).

### Antioxidant capacity in the liver and spleen

**Fig. 4:**
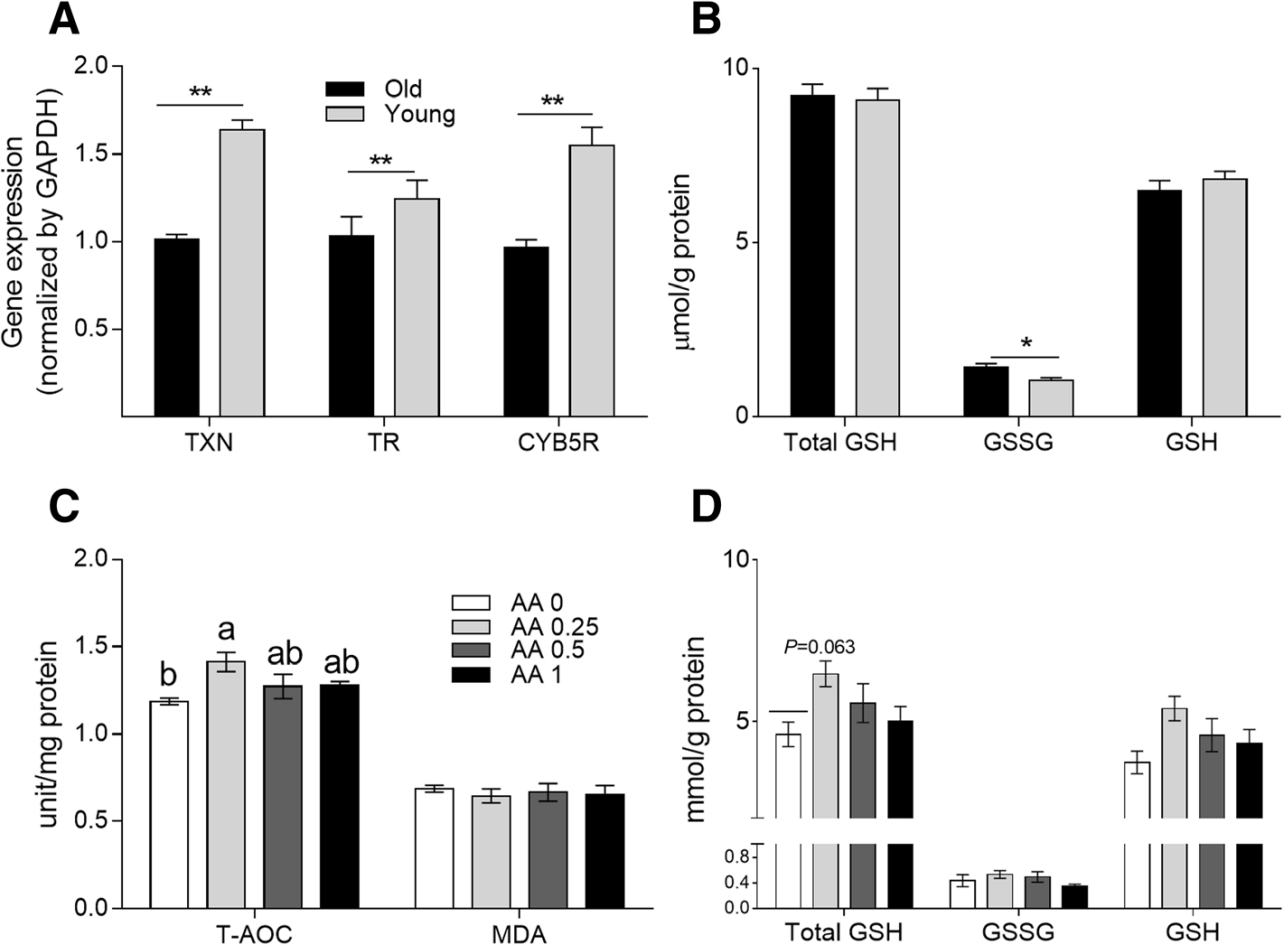
Antioxidant status and immunity of laying hens. **a** Gene expression levels of thioredoxin (*TXN*), thioredoxin reductase (*TXNR*), and cytochrome b5 reductase (*CYB5R*) and **b** GSH/GSSG contents in the liver of the old and young hens in Exp. 1. *means *P* < 0.05; **means *P* < 0.01. **c** Effects of dietary supplementation of AA on T-AOC and malonaldehyde (MDA) level and **d** Total GSH, GSSG, and GSH concentrations in the liver of old laying hens in Exp. 2. Within each panel, means without a common letter differ at *P* < 0.05

As shown in Fig. 4a, in Exp. 1, the young hens showed higher *TXN*, *TXNR*, and *CYb5R* gene expression levels (*P* < 0.05) in the liver than did the old hens. Elevated GSSG concentrations (*P* < 0.05) were found in the livers of old hens (shown in Fig. 4b). In Exp. 2, old hens on diets supplemented with 0.25 g AA/kg had increased T-AOC and total GSH contents in the liver relative to the control hens (*P* < 0.05 and *P* = 0.06, respectively). However, AA supplemented at 0.5 or 1 g/kg diet had no significant effect on T-AOC (*P* > 0.05). Similarly, no significant differences in malondialdehyde levels among the four treatments (*P* > 0.05) were observed (as shown in Fig. 4c and d).

### Production performance and egg quality in Exp. 2

The effects of dietary AA supplementation on the performance of laying hens are shown in Table 3. No significant difference in production performance or egg quality was observed among treatments over the experimental period (*P* > 0.05).

**Table 3:** Effects of supplementation with AA on performance and egg quality in old laying hens

Items	Treatment, g AA/kg diet				SEM^b^	*P*-value		
	0	0.25	0.5	1		Main effect	Linear	Quadratic
Performance								
Laying rate, %	86.0	86.5	86.0	81.9	0.80	0.144	0.075	0.138
Egg weight, g	65.4	64.4	64.6	65.0	0.28	0.597	0.674	0.222
ADFI^a^, g	117	118	117	119	0.6	0.672	0.492	0.611
FCR	2.13	2.13	2.12	2.20	0.023	0.170	0.097	0.126
Egg quality								
Eggshell thickness, mm	0.34	0.34	0.34	0.34	0.002	0.107	0.106	0.798
Eggshell strength, kg/cm^3^	3.10	3.14	3.03	3.01	0.031	0.773	0.382	0.734
Albumen height, mm	6.39	6.32	6.92	6.39	0.079	0.738	0.761	0.598
Haugh unit	76.7	77.0	76.6	76.4	0.60	0.988	0.807	0.825
Yolk colour	6.82	6.80	6.83	6.85	0.029	0.959	0.653	0.807
Eggshell portion, %	9.88	9.91	9.81	9.73	0.059	0.748	0.322	0.650

Data represent the mean of 30 cages (three old laying hens per cage) per treatment

^a^*ADFI* average daily feed intake, *FCR* feed conversion rate

^b^The SEM values represent the overall standard errors of the mean in each row

**Fig. 5:**
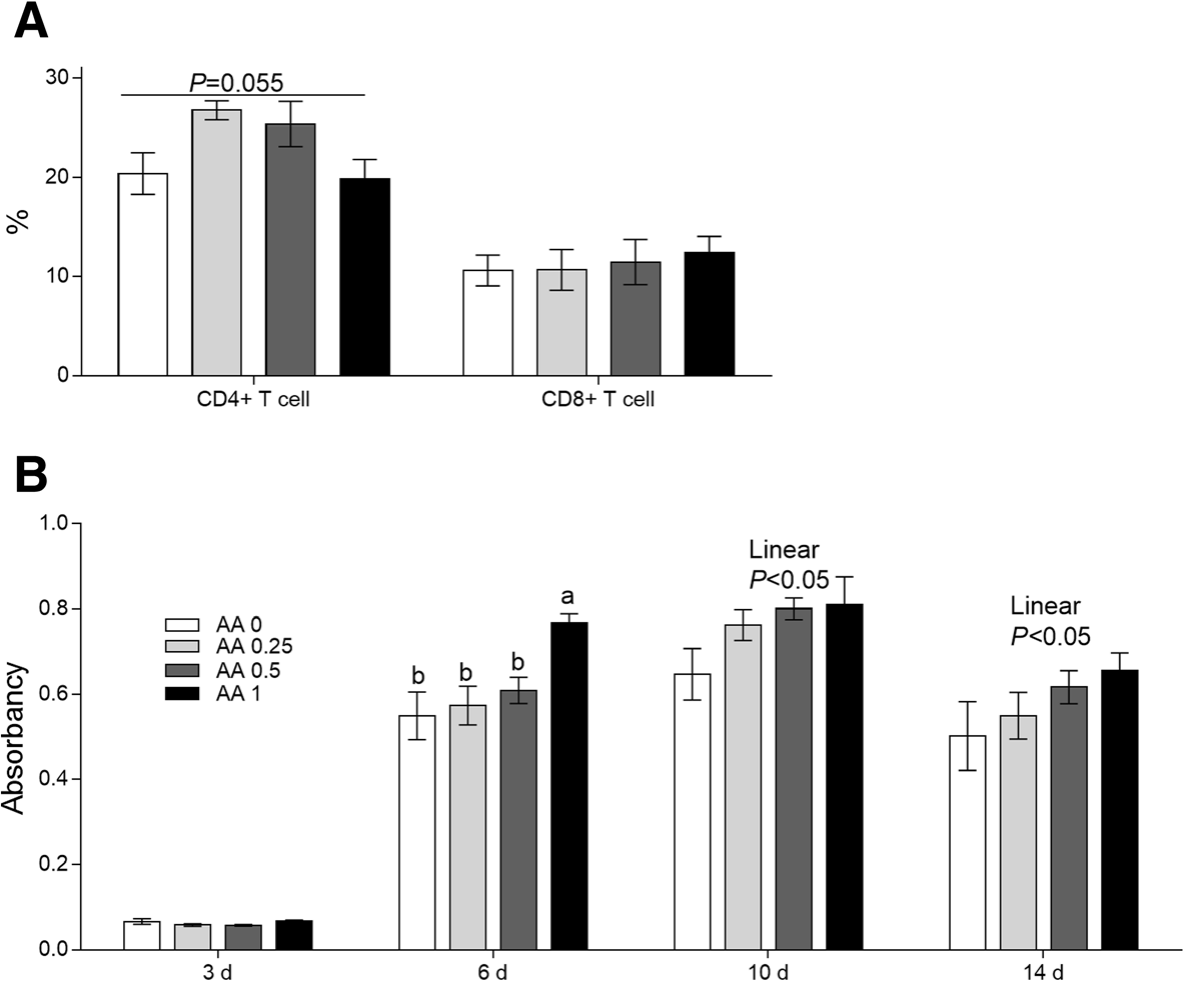
Effects of dietary AA on the portion of T lymphocytes **a** and the IgG levels at 3, 6, 10, and 14 d post-immunity with BSA **b** in serum in Exp. 2. Within each panel, means without a common letter differ at *P* < 0.05

Serum immunoglobulin G against bovine serum albumin and the portion of CD4^+^ and CD8^+^ T lymphocytes. As shown in Fig. 5b, at d 6 after immunization with BSA, dietary supplementation with 1 g AA/kg diet significantly increased serum IgG level (*P* < 0.05) relative to the levels in the control and other treatment groups. Based on the observations at d 10 and d 14 after immunization with BSA, IgG level increased linearly with increasing AA amount (*P* < 0.05).

An increasing trend (*P* = 0.06) in CD4^+^ T lymphocyte numbers was recorded after the addition of the 0.25 and 0.5 g AA/kg diets for the old laying hens. However, the portion of CD8^+^ T lymphocytes were not influenced among all the treatment groups, and these results are presented in Fig. 5a.

## Discussion

Ascorbic acid is an essential nutrient for poultry to maintain optimal production and resist the effects of various stresses. It is generally believed that poultry can synthesize AA in kidney with the action of the GLO partially meeting the requirement [7, 22]. An interesting finding of the present study was that gene expression and GLO activity in the kidney did not differ between old (over 75 weeks of age) and young layers (35–39 weeks of age). In addition, in this study, GLO enzyme activities were detected in the liver (the activities of GLO in the liver account for only 0.5–0.7% of that in the kidney), suggesting that laying hens have the capacity to synthesize small quantities of AA in the liver. This finding contradicts reports that AA cannot be synthesized in the poultry liver [18]. The inconsistent results might be related to the different methods of AA detection. The HPLC method used in this experiment is more sensitive and accurate in detecting low levels of AA than is the spectrophotometric assay used in previous reports [18, 23, 24]. In addition, GLO enzyme activity can be influenced by multiple factors, including the environment, feed, age, strain of poultry, and the methods of sample acquisition and storage [25, 26]. However, the very low activity of GLO in the liver contributes minimally to overall AA synthesis in poultry.

Endogenous synthesis, absorption from the diet and reabsorption from the urine are the main mechanisms by which poultry meet the demand for AA [27, 28]. The present work indicated that exogenous AA via the diet decreases GLO activity in the kidney and reduces the endogenous synthesis of AA. This suggests the presence of an internal feedback mechanism of regulation in hens whereby dietary supplementation of AA reduces the requirement for endogenous AA production, thereby reducing AA synthesis in vivo by decreasing GLO activity. The suggested mechanism is consistent with Hooper et al. [25], who reported that the addition of 1 g AA/kg diet into broiler diets decreased GLO activity in the kidney.

It has been reported that SVCT1 is primarily distributed in the epithelial cells of some tissues, such as intestine and kidney, participating in whole-body homeostasis and metabolic requirements through intestinal absorption and renal tubular reabsorption of AA [8, 22]. This study showed that the mRNA expression of the transporter *SVCT1* was greater in the ileum than in the duodenum and jejunum, indicating that the main absorption site of AA is the ileum. However, the transporter results are inconsistent with our hypothesis that the expression of *SVCT1* in the ileum and kidney and *SVCT2* in the kidney are higher in old hens than in young hens. Our results suggest that compared with young layers, old layers had greater capacities to absorb AA from the digestive tract and to reabsorb AA from their urine. Through the increased abilities to absorb and reabsorb AA, rather than through endogenous synthesis, old layers improved AA retention in some tissues. This phenomenon might be an intrinsic physiological mechanism to regulate AA metabolism in old layers.

In various tissues, such as kidney, liver, spleen, and various glands, AA plays important roles in reducing the accumulation of peroxidant compounds and maintaining the physiological function in organs [29]. Moreover, prior studies have documented that the distribution and retention of AA in organs vary with animal age [30]. The increased AA contents in the liver and spleen in Exp. 1 might reflect a defence mechanism of old hens in responding to stresses, with the hens enhancing the antioxidant capabilities of immune organs. The shell gland plays an important role in the production of the egg shell. The higher AA content in the egg-shell glands of young layers than in those of old layers enhances antioxidant status, which is favourable for eggshell quality. In Exp. 2, dietary supplementation of exogenous AA elevated the concentration of AA in the spleen, suggesting the presence of enhanced antioxidant and immune capacities. The spleen plays vital roles in the immune systems of old laying hens because of the age-related involution of the thymus [31]. It has been documented that AA can promote the differentiation of B cells and IgM production [32], scavenge free radicals produced by macrophages during the bacteria-fighting process [33], and accelerate the maturation of T lymphocytes [34]. Thus, the increases in the AA and DHA concentrations in the spleen resulting from dietary AA supplementation might contribute to the elevated CD4^+^ T lymphocyte numbers and the increased anti-BSA IgG concentrations in the sera of the 78-week-old laying hens. CD40 ligand, usually expressed on the surface of CD4^+^ T lymphocytes, plays vital roles in activating B cells through binding to CD40, which is expressed on the surfaces of B cells [35]. There is an increasing trend in the CD4^+^ T lymphocyte numbers in AA supplemental treatments. Moreover, the elevated CD4^+^ T lymphocyte numbers might have greater capacity to activate B cells, resulting in greater levels of anti-BSA IgG production in the serum. The results of increased IgG levels in this study are in agreement with the observation that AA can promote antibody production as part of the immune response [32]. Thus, the results indicate that dietary supplementation with AA can enhance humoral immunity in old laying hens. Similarly, supplementation of 1 g AA/kg diet has been found to reverse the immunosuppression caused by IBDV vaccination and improve the humoral and cellular immune responses of chickens [36]. Increased IgG levels are important in protecting old laying hens from bacterial invasion and other stresses [32, 34].

Unexpectedly, inclusion of AA in the diet of 78 weeks old layers did not increase production performance but did enhance antioxidant status and immunity. Dietary addition of 0.25 g AA/kg diet increased the contents of T-AOC and the total GSH in the liver of old laying hens. Excessive supplementation (above 1 g/kg diet) may have negative-feedback inhibition on the secretion of endogenous antioxidant enzymes such as T-AOC and GSH. However, the increased tendency of total GSH and increased T-AOC levels in the liver of old laying hens following dietary supplementation of AA indicated an elevated antioxidant status of these hens. Similarly, Wang et al. [3] reported that inclusion of AA in duckling diets significantly increased antioxidant status. The enhanced antioxidant capacity of the old laying hens likely decreased their vulnerability, disability, and risk of mortality. The current study was limited by the small number (360) of laying hens and the short trial period due to restrictions imposed by the research facility. The limited experimental period might have led to the lack of an observed effect on production performance. It is well known that hydrogen peroxide, formed during the period when GLO catalyses the last step of AA synthesis, would be metabolized at the expense of antioxidants such as catalase and GSH [37]. Exogenous supplementation of AA can reduce the amount of AA synthesised in vivo, thus decreasing the accumulation of hydrogen peroxide, contributing to the improved antioxidant status. This effect was consistent with our finding that the old laying hens had improved immune and antioxidant statuses but decreased GLO enzyme activity due to the incorporation of AA in the diet. Therefore, it would be feasible to add dietary AA for the improvement of the health conditions of laying hens. In practice, the nutrient requirement of AA for laying hens varies greatly due to variation in feeding conditions. Further studies of the effects of AA on old laying hens and the optimal dosages under different rearing conditions should be conducted to confirm the findings of this study.

## Conclusions

**Fig. 6:**
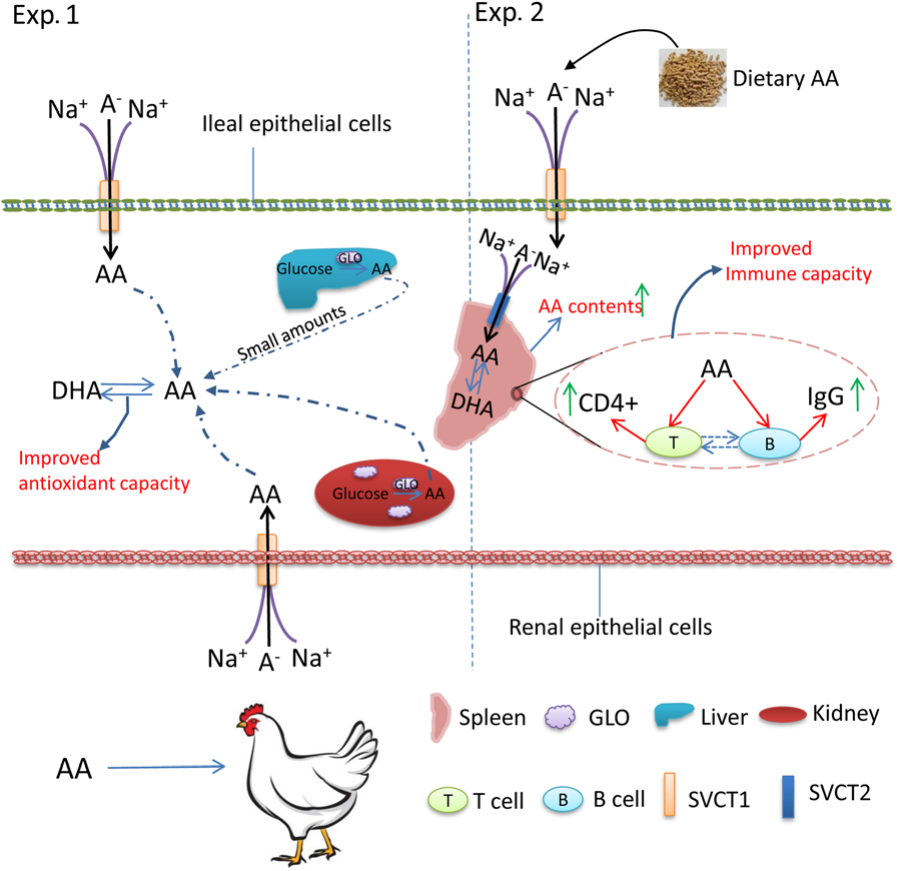
The mechanisms of AA metabolism and transport in old laying hens. In Exp. 1, higher absorption and reabsorption capacities were found in 75 weeks old old laying hens compared with the 35 weeks old young hens. Small amounts of AA synthesized in liver were found. The four sources of AA (intestinal absorption, renal re-absorption, and synthesis in kidney (primary) and liver (small quality)) facilitate the higher AA contents in the liver and spleen and the greater antioxidant capacity of the old laying hens. In Exp. 2, supplementation with exogenous AA contributed to the enhanced retention of AA in the spleen and the increased portion of CD4^+^ T lymphocytes and IgG concentration in the sera of 78 weeks old laying hens, suggesting a strengthened immune status. Dietary supplementation of AA improved antioxidant capacity and immune status, indicating the enhanced health condition of the old laying hens

In summary, laying hens can synthesize a small quantity of AA in the liver. By up-regulating the expression levels of transporter genes, the old laying hens enhanced their capacities for intestinal AA absorption and renal AA reabsorption, which led to greater concentrations of AA in immunological tissues. Dietary supplementation of AA improved the health of the old laying hens by increasing their antioxidant status and immunity, as evidenced by the increased activities of antioxidant enzymes, increased IgG levels, and increased AA retention in the spleen (Fig. 6). Considering all of the investigated variables, the optimum amount of dietary AA supplementation for laying hens appears to be approximately 0.25 g AA/kg diet.

## Abbreviations

AA: Ascorbic acid
BSA: Bovine serum antigen
CYB5R: Cytochrome b5 reductase
DHA: Dehydroascorbic acid
GLO: L-gulonolactone oxidase
MDA: Malonaldehyde
PB: Sodium phosphate buffer
SVCTs: Sodium-dependent vitamin C transporters
T-AOC: Total antioxidant capacity
TXN: Thioredoxin
TXNR: Thioredoxin reductase

## References

[1] Molnar A, Maertens L, Ampe B, Buyse J, Zoons J, Delezie E (2017). Supplementation of fine and coarse limestone in different ratios in a split feeding system: effects on performance, egg quality, and bone strength in old laying hens. Poult Sci.

[2] Molnar A, Maertens L, Ampe B, Buyse J, Kempen I, Zoons J (2016). Changes in egg quality traits during the last phase of production: is there potential for an extended laying cycle?. Br Poult Sci.

[3] Wang A, Xie F, Wang YH, Wu JL (2011). Effects of vitamin C supplementation on growth performance and antioxidant status of layer ducklings. J Anim Physiol Anim Nutr.

[4] Keshavarz K (1996). The effect of different levels of vitamin C and cholecalciferol with adequate or marginal levels of dietary calcium on performance and eggshell quality of laying hens. Poult Sci.

[5] Zhou Q, Wang L, Wang H, Xie F, Wang T (2012). Effect of dietary vitamin C on the growth performance and innate immunity of juvenile cobia (Rachycentron canadum). Fish Shellfish Immunol.

[6] Monacelli F, Acquarone E, Giannotti C, Borghi R, Nencioni A (2017). Vitamin C, aging and Alzheimer's disease. Nutrients.

[7] Chaudhuri CR, Chatterjee IB (1969). Ascorbic acid synthesis in birds. Science.

[8] Corti A, Casini AF, Pompella A (2010). Cellular pathways for transport and efflux of ascorbate and dehydroascorbate. Arch Biochem Biophys.

[9] Young JI, Zuchner S, Wang G (2015). Regulation of the epigenome by vitamin C. Annu Rev Nutr.

[10] Ahmed W, Ahmad S, Ahsan-ul-haq KZ (2008). Response of laying hens to vitamin C supplementation through drinking water under sub-tropical conditions. Avian Biol Res.

[11] Wang JP, He KR, Ding XM, Luo YH, Bai SP, Zeng QF (2016). Effect of dietary vanadium and vitamin C on egg quality and antioxidant status in laying hens. J Anim Physiol Anim Nutr.

[12] Creel LH, Maurice DV, Lightsey SF, Grimes LW (2001). Stability of dietary ascorbic acid and the effect of supplementation on reproductive performance of broiler breeder chickens. Br Poult Sci.

[13] Alizadeh M, Munyaka P, Yitbarek A, Echeverry H, Rodriguez-Lecompte JC (2017). Maternal antibody decay and antibody-mediated immune responses in chicken pullets fed prebiotics and synbiotics. Poult Sci.

[14] Fan H, Lv Z, Gan L, Guo Y (2018). Transcriptomics-related mechanisms of supplementing laying broiler breeder hens with dietary Daidzein to improve the immune function and growth performance of offspring. J Agric Food Chem.

[15] Robitaille L, Hoffer LJ (2016). A simple method for plasma total vitamin C analysis suitable for routine clinical laboratory use. Nutr J.

[16] Amano A, Aigaki T, Maruyama N, Ishigami A (2010). Ascorbic acid depletion enhances expression of the sodium-dependent vitamin C transporters, SVCT1 and SVCT2, and uptake of ascorbic acid in livers of SMP30/GNL knockout mice. Arch Biochem Biophys.

[17] Chatterjee IB, Chatterjee GC, Ghosh NC, Ghosh JJ, Guha BC (1960). Biological synthesis of L-ascorbic acid in animal tissues: conversion of L-gulonolactone into L-ascorbic acid. Biochem J.

[18] Hooper CL, Maurice DV, Lightsey SF, Toler JE (2000). Factors affecting ascorbic acid biosynthesis in chickens. I. Adaptation of an assay and the effect of age, sex, and food deprivation. J Anim Physiol Anim Nutr.

[19] Ching S, Mahan DC, Moreau R, Dabrowski K (2003). Modification of analytical procedures for determining vitamin C enzyme (L-gulonolactone oxidase) activity in swine liver. J Nutr Biochem.

[20] Livak KJ, Schmittgen TD (2001). Analysis of relative gene expression data using real-time quantitative PCR and the 2(−Delta Delta C(T)) method. Methods.

[21] Li C, Guo S, Zhang M, Gao J, Guo Y (2015). DNA methylation and histone modification patterns during the late embryonic and early postnatal development of chickens. Poult Sci.

[22] Figueroa-Méndez R, Rivas-Arancibia S (2015). Vitamin C in health and disease: its role in the metabolism of cells and redox state in the brain. Front Physiol.

[23] Zannoni V, Lynch M, Goldstein S, Sato P (1974). A rapid micromethod for the determination of ascorbic acid in plasma and tissues. Biochem Med.

[24] Dabrowski K, Hinterleitner S (1989). Applications of a simultaneous assay of ascorbic acid, dehydroascorbic acid and ascorbic sulphate in biological materials. Analyst.

[25] Hooper CL, Maurice DV, Lightsey SF, Toler JE (2002). Factors affecting ascorbic acid (AsA) biosynthesis in chickens. II.Effect of dietary AsA and strain of chicken. J Anim Physiol Anim Nutr.

[26] Maurice DV, Lightsey SF, Abudabos A, Toler JE (2002). Factors affecting ascorbic acid biosynthesis in chickens. III. Effectof dietary fluoride on L-gulonolactone oxidase activity and tissueascorbic acid (AsA) concentration. J Anim Physiol Anim Nutr.

[27] Kuo S-M, Tan C-H, Dragan M, Wilson JX (2005). Endotoxin increases ascorbate recycling and concentration in mouse liver. J Nutr.

[28] Corpe CP, Tu H, Eck P, Wang J, Faulhaber-Walter R, Schnermann J (2010). Vitamin C transporter Slc23a1 links renal reabsorption, vitamin C tissue accumulation, and perinatal survival in mice. J Clin Invest.

[29] Aumailley L, Warren A, Garand C, Dubois MJ, Paquet ER, Le Couteur DG (2016). Vitamin C modulates the metabolic and cytokine profiles, alleviates hepatic endoplasmic reticulum stress, and increases the life span of Gulo−/− mice. Aging.

[30] Iwama M, Amano A, Shimokado K, Maruyama N, Ishigami A (2012). Ascorbic acid levels in various tissues, plasma and urine of mice during aging. J Nutr Sci Vitaminol.

[31] Uchio R, Hirose Y, Murosaki S, Yamamoto Y, Ishigami A (2015). High dietary intake of vitamin C suppresses age-related thymic atrophy and contributes to the maintenance of immune cells in vitamin C-deficient senescence marker protein-30 knockout mice. Br J Nutr.

[32] Ichiyama K, Mitsuzumi H, Zhong M, Tai A, Tsuchioka A, Kawai S (2009). Promotion of IL-4- and IL-5-dependent differentiation of anti-mu-primed B cells by ascorbic acid 2-glucoside. Immunol Lett.

[33] Mohammed BM, Fisher BJ, Huynh QK, Wijesinghe DS, Chalfant CE, Brophy DF (2014). Resolution of sterile inflammation: role for vitamin C. Mediat Inflamm.

[34] Manning J, Mitchell B, Appadurai DA, Shakya A, Pierce LJ, Wang H (2013). Vitamin C promotes maturation of T-cells. Antioxid Redox Signal.

[35] Chen Q, Ross AC (2007). Retinoic acid promotes mouse splenic B cell surface IgG expression and maturation stimulated by CD40 and IL-4. Cell Immunol.

[36] Wu CC, D T, Lin TL (2000). Effect of ascorbic acid supplementation on the immune response of chickens vaccinated and challenged with infectious bursal disease virus. Vet Immunol Immunopathol.

[37] Linster CL, Van Schaftingen E, Vitamin C (2007). Biosynthesis, recycling and degradation in mammals. FEBS J.

